# Renal Bone Disease Among Patients With ESRD

**DOI:** 10.5812/numonthly.9556

**Published:** 2013-06-05

**Authors:** Seyed Seifollah Beladi Mousavi, Hossein Saghafi

**Affiliations:** 1 Department of Internal Medicine, Faculty of Medicine, Jundishapour University of Medical Sciences, Ahvaz, IR Iran; 2 Department of Internal Medicine, Qom University of Medical Sciences, Qom, IR Iran

**Keywords:** Kidney Failure, Chronic, Renal Osteodystrophy

*Dear Editor,*

With interest, we read the article by Sidy Secket et al. on the mineral and bone disease in black African hemodialysis patients: A report from Senegal. The authors evaluated 118 end-stage renal disease (ESRD) patients under hemodialysis and showed that chronic kidney disease related mineral and bone disease (CKD-MBD) are frequent in Senegalese hemodialysis patients with prevalence of 66.9 % and hyperparathyroidism is the most commonplace disorder followed by adynamic bone disease and osteomalacia (1).

Unfortunately CKD-MBD is also common in other developed and developing countries and this will cause an enormous financial burden for health-care systems (2, 3). There appears to be an increased risk of all-cause and cardiovascular mortality among ESRD patients with disorders of mineral metabolism (4).

Although, renal bone and mineral disease are progressive lesions among patients with CKD, symptoms and/or signs due to the various CKD-MBD, such as fractures, bone and muscle pain, generally do not occur until the patient is undergoing maintenance dialysis (2).

Five main types of renal bone and mineral disease can be seen among patients with CKD. These are 1) osteitis fibrosa cystic, in which bone turnover is increased due to secondary hyperparathyroidism, 2) adynamic or aplastic bone disease, in which bone turnover is low due primarily to excessive suppression of the parathyroid glands and in some cases due to aluminum deposition, 3) osteomalacia, in which bone turnover is also low in combination with a marked increase in the volume of unmineralized bone, most current cases result from aluminum deposition in bone among patients who used aluminum-containing antacids as phosphate binders, 4) mixed renal osteodystrophy, a mixture of the first and three type, in which elements of both high and low bone turnover may be observed and presented as marrow fibrosis and increased unmineralized osteoid, and 5- beta 2-microglobulin-associated amyloid deposit’s bone disease in which occur in patients on long-term dialysis and characterized by bone cysts (5-8).

It appears that the prevalence of the different types of bone disease is considerably different between developed and developing countries (1, 9). For example in the result of Sidy Secket et al. study in Senegal a developing country, osteitis fibrosa cystica due to secondary hyperparathyroidism is the most common type of renal osteodystrophy which occurred in 55 patients with ESRD followed by adynamic bone disease in which occurred in 21 patients and osteomalacia in which occurred in 1 patient (1).

However in developed countries, the prevalence of secondary hyperparathyroidism among dialysis patients has markedly decreased compared to last few decades and at present, the most common disorder is non-aluminum-induced adynamic bone disease, with osteitis fibrosa due to secondary hyperparathyroidism, osteomalacia, and mixed disease less frequently observed (10-13).

High frequency of non-aluminum-induced adynamic bone disease in developed countries can be explained by multiple factors, including changes in therapeutic strategies such as the increased and earlier use of vitamin D analogs and calcium-containing phosphate binders (14, 15).

Patients' demographics (older and increased number of diabetic patients) may also be implicated in the increasing number of patients with adynamic bone disease among these countries. For example in a bone biopsy study in 84 patients with stage 5 CKD not yet on maintenance dialysis, adynamic bone disease was the most common type of renal bone disease among patients with diabetes mellitus (14).

We have a methodological comment on the study by Sidy Secket et al. The authors did not perform bone biopsy for diagnosing the type of renal bone disease and it is a limited factor for the study (1). Bone biopsy is the gold standard for establishing the various types of renal osteodystrophy and there is some new evidence that biochemical parameters such as is serum intact PTH level is not sufficiently accurate for establishing the type of renal bone disease among patients with CKD (16).

The discrepancy between biochemical parameters and the type of renal bone disease was shown in retrospective study of Gal-Moscovici A et al. They compared serum intact PTH levels with results from bone biopsy and showed no correlation between bone biopsy results and PTH levels in 150 to 500 pg/mL and 500 to 1,200 pg/mL. However a significant correlation is observed in this study between adynamic bone disease and PTH levels less than 150 pg/mL (16).
